# Prevalence of respiratory symptoms and spirometric changes among non-smoker male wood workers

**DOI:** 10.1371/journal.pone.0224860

**Published:** 2020-03-18

**Authors:** Davood K. Hosseini, Vahab Malekshahi Nejad, Haiying Sun, Hanieh K. Hosseini, Seyyed Hassan Adeli, Tian Wang

**Affiliations:** 1 Department of Medicine, Stanford University School of Medicine, Stanford, CA, United States of America; 2 Department of Pulmonary Medicine, Qom University of Medical Sciences, Qom, Iran; 3 Department of Otorhinolaryngology, Union Hospital, Tongji Medical College, Huazhong University of Science and Technology, Wuhan, Hubei, China; 4 Department of Otolaryngology-Head and Neck Surgery, Stanford University School of Medicine, Stanford, CA, United States of America; 5 Department of Otolaryngology-Head and Neck Surgery, The Second Xiangya Hospital, Central South University, Changsha, Hunan Province, China; Telethon Institute for Child Health Research, AUSTRALIA

## Abstract

**Objective:**

To assess the effects of workplace exposure to hardwood dust on lung function and determine a prevalence of respiratory symptoms among wood workers.

**Study design:**

Cross-sectional observational study.

**Setting:**

Tertiary referral center.

**Subjects and methods:**

Two hundred seventy-six, non-smoker male wood workers and equal number of non-smoker male office workers, referred to pulmonology clinic included in this study. Evaluation of study participants included completion of a questionnaire regarding respiratory symptoms and baseline spirometry was measured according to the actual recommendations.

**Results:**

Respiratory symptoms including cough, phlegm, chest tightness, and wheezing were significantly higher in wood workers than office workers (40.2% versus 29.3% for cough, p = 0.0073; 40.6% versus 23.6% for phlegm, p<0.0001; 38.0% versus 23.1% for chest tightness, p = 0.0001; 25.3% versus 14.5% for wheezing, p = 0.0014).

No statistically significant differences were observed for Dyspnea, and upper respiratory tract symptoms among wood workers compared to office workers. While wood workers were more likely to require spirometry test than office workers (21.4% versus 5.4%, p<0.001) the obstructive changes were more prevalent on spirometry test in wood workers (71.4% obstructive pattern versus 28.6% restrictive pattern). Spirometry test revealed the mean values of FEV_1_ and FEV_1_/FVC ratio were significantly lower in the wood workers, compared to their mean values in the control group.

**Conclusion:**

Respiratory symptoms associated with work, are more prevalent among wood workers than office workers. Our data revealed that workplace exposure to hardwood dust may compromise respiratory function, indicating the importance and the need for optimizing preventive measures in workplace to protect the respiratory health among exposed workers. Obstructive changes on pulmonary function test is a dominant pathologic pattern in pulmonary function test among wood workers. Further investigation is required by current available tools such as nasal cytology to detect influence of wood dust exposure on the upper respiratory airway.

## Introduction

Occupational lung disease comprises a broad spectrum of disorders as a result of inhalation or ingestion of noxious chemicals or dust particles, and despite governmental safety standard regulations, it remains one of the most common work-related injuries worldwide[1, 2].

**Fig 1:**
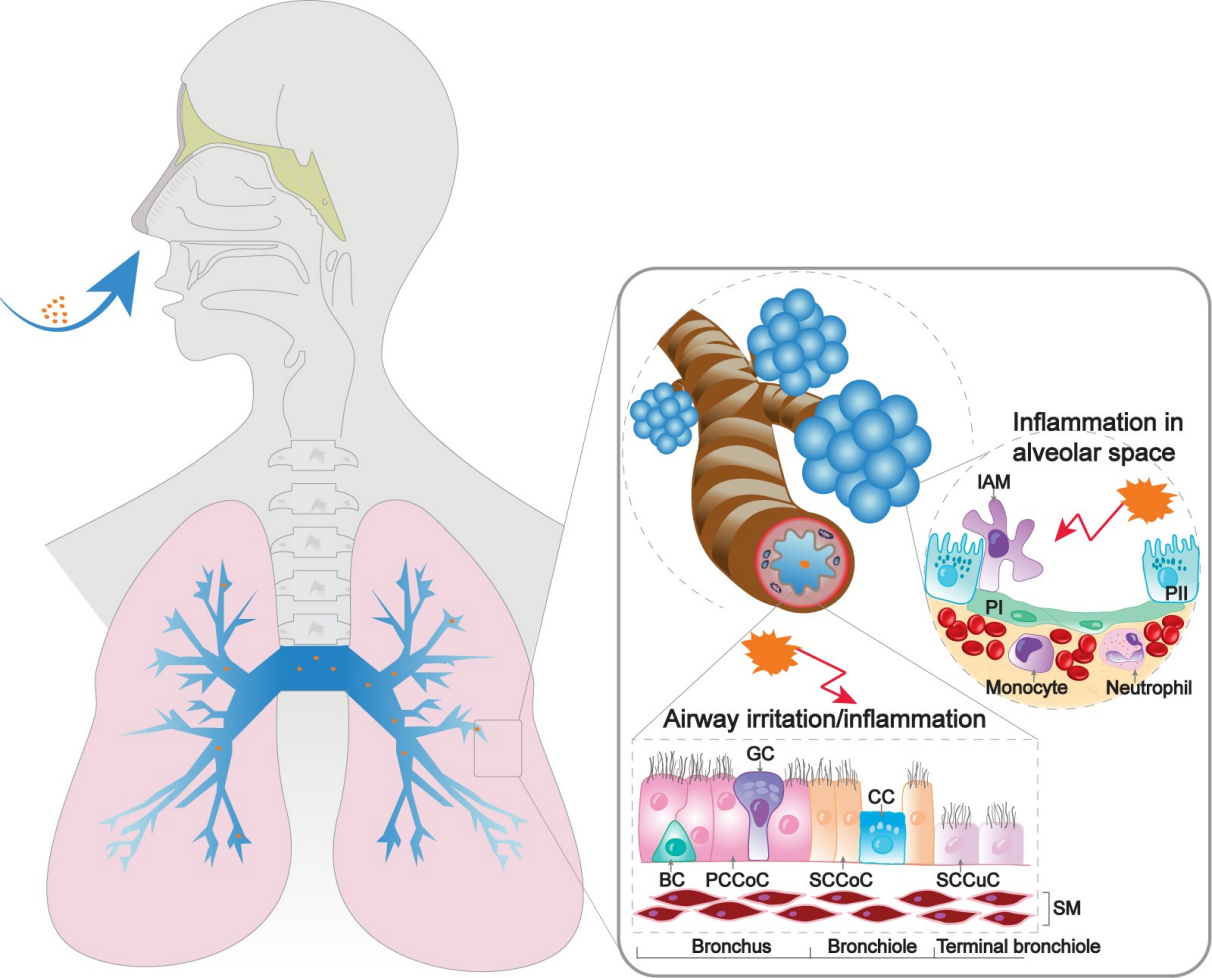
Schematic view of respiratory system homeostasis in response to dust and chemical exposure. Occupational exposure to dust and chemical leads to irritation and initiation of inflammatory responses in the host respiratory system, that require the engagement of different regulatory cellular pathways. Alveolar macrophages demonstrate the pro-inflammatory and anti-inflammatory properties that contribute to the pulmonary hemostasis. IAM: intra alveolar macrophage, PI: type one pneumocyte, PII: type two pneumocyte, BC: basal cell, PCCoC: pseudostratified ciliated columnal epithelial cell, SCCoC: simple ciliated columnar epithelial cell, SCCuC: simple ciliated cuboidal epithelial cell, GC: goblet cell, CC: Clara cell, SM: smooth muscle.

Factors predisposing industrial workers to respiratory diseases include heavy, short or prolonged exposure to gases, chemicals, and dust. Occupational exposure to dust and chemical leads to irritation and initiation of inflammatory responses in the host respiratory system, that require the engagement of different regulatory cellular pathways. Alveolar macrophages demonstrate the pro-inflammatory and anti-inflammatory properties that contribute to the pulmonary hemostasis[3] (Fig 1).

As an example prolong unprotected inhalation of wood smokes leads to series of respiratory symptoms including nasal congestion, cough, chest tightness and wheezing[4]. On the other hand, industrial wood operations like peeling, slicing, milling, drilling, and sawing give out fine wood dust which becomes airborne. This contains wide-ranging chemicals, including cellulose, hemicelluloses, lignin as well as extraneous materials and may result in respiratory health chanllenges[5, 6]. Non-smoker male wood workers exposed to natural hardwood dust had significantly lower selected respiratory parameters than their counterparts working in the offices[7]. In 2008 Schlunssen et al. reported wood dust exposure might causes respiratory symptoms in sawmill workers despite being exposed to relatively low levels of chemical particles[8]. This on one hand, devoid these workers from safety and welfare facilities, and on the other hand puzzle the policymakers to estimate the burden of the health problem.

Nowadays, the importance of preventive measures widely accepted, however the effect of occupational exposure to airborne agents in the development of lung disease is still not fully understood; hence it is ripe for research and requires further investigations.

This study compared the lung function and 12 months period prevalence of respiratory symptoms between a group of non-smoker male wood workers exposed to natural hardwood dust and smoke and a group of unexposed non-smoker male office workers.

## Material and methods

A cross-sectional observational study was carried out among male wood workers, and equal number of male office workers whom referred to pulmonology clinic, between 2008 and 2018.

Institutional review board approval was obtained from the Qom University of Medical Science. Male woodworkers with a variety of respiratory complains were included, although patients with history of smoking or chronic respiratory disease diagnosed by a physician were excluded. Informed consent from participants obtained before entering the study. A 12 months period prevalence of respiratory symptoms and mean values of spirometric parameters was compared.

### Respiratory symptoms questionnaire

All participants allowed to fill the questionnaire regarding demographic information, past medical history, and respiratory symptoms.

Respiratory symptoms in the last 12 months, including wheezing, cough, phlegm, chest tightness, dyspnea, nasal congestion, rhinorrhea, and sore throat were recorded. [9, 10].

The questionnaire was translated into Farsi and back translated into English (S1 and S2 Forms). The study team was particularly trained to make sure that the participants were able to truly comprehend the meaning of all questions.

### Lung function measurement

All Spirometry measurements were performed by trained pulmonology clinical technician. Spirometry test was completed using Spirolab II (Spirolab II, SDI, USA) auto-calibrating device. The original commercial protocol was consistently utilized. For validation and accuracy of testing, equipment quality control, including regular calibration checks before testing was performed, according to the American Thoracic Society recommendations[11].

Forced expiratory volume in the first second (FEV_1_), forced vital capacity (FVC), and the ratio (FEV_1_/FVC) standardized for age, gender, height, body surface area, and duration of exposure were assessed. There were no subjects in neither group with contraindication for spirometry.

Microsoft Excel (Microsoft) and GraphPad Prism 8.0 software (GraphPad) were used to run statistical analyses. Two-tailed, unpaired student’s t-tests were used to determine statistical significance. p<0.05 was considered to be significant.

## Results

Of the 420 male woodworker who initially assessed, 144 patients were excluded due to history of smoking or chronic respiratory disease diagnosed by a physician (i.e. lung cancer, COPD, asthma, idiopathic pulmonary fibrosis, etc.). An equal group of non-smoker male office workers matched to woodworkers by age and duration of employment at actual workplace was included as a control (n = 276) (Table 1).

**Table 1:** Demographics of the study subjects.

Variable	Wood workers (n = 276)	Office workers (n = 276)
Age (years)	46.36 ± 7.72	49.18 ± 10.90
BMI (kg/m^2^)	25.28 ± 4.08	25.12 ± 3.44
Duration of employment < 15 years	172 (62.32%)	182 (65.94%)
Duration of employment ≥ 15 years	104 (37.68%)	94 (34.06%)

Values shown for continuous variables are mean ± SD; number and percentage for categorical variables. BMI: body mass index; kg: kilogram; m: meter.

Reviewing patient medical records revealed that ischemic heart disease found more prevalent among office workers than wood workers (7.2% versus 3.3%, p = 0.0366) (Table 2).

**Table 2:** Past medical history of the study subjects.

Medical history	Wood workers (n = 276)	Office workers (n = 276)	P-value*
Diabetes mellitus type II	12 (4.3%)	18 (6.5%)	0.2600
Arterial hypertension	10 (3.6%)	12 (4.3%)	0.6634
Dyslipidemia	8 (2.9%)	5 (1.8%)	0.3998
Ischemic heart disease	9 (3.3%)	20 (7.2%)	0.0366
Musculoskeletal disorders	13 (4.7%)	5 (1.8%)	0.0552

* Chi-square test.

The prevalence of respiratory symptoms analyzed (in the absent of pulmonary infection or common cold):

### Upper respiratory tract symptoms

**Fig 2:**
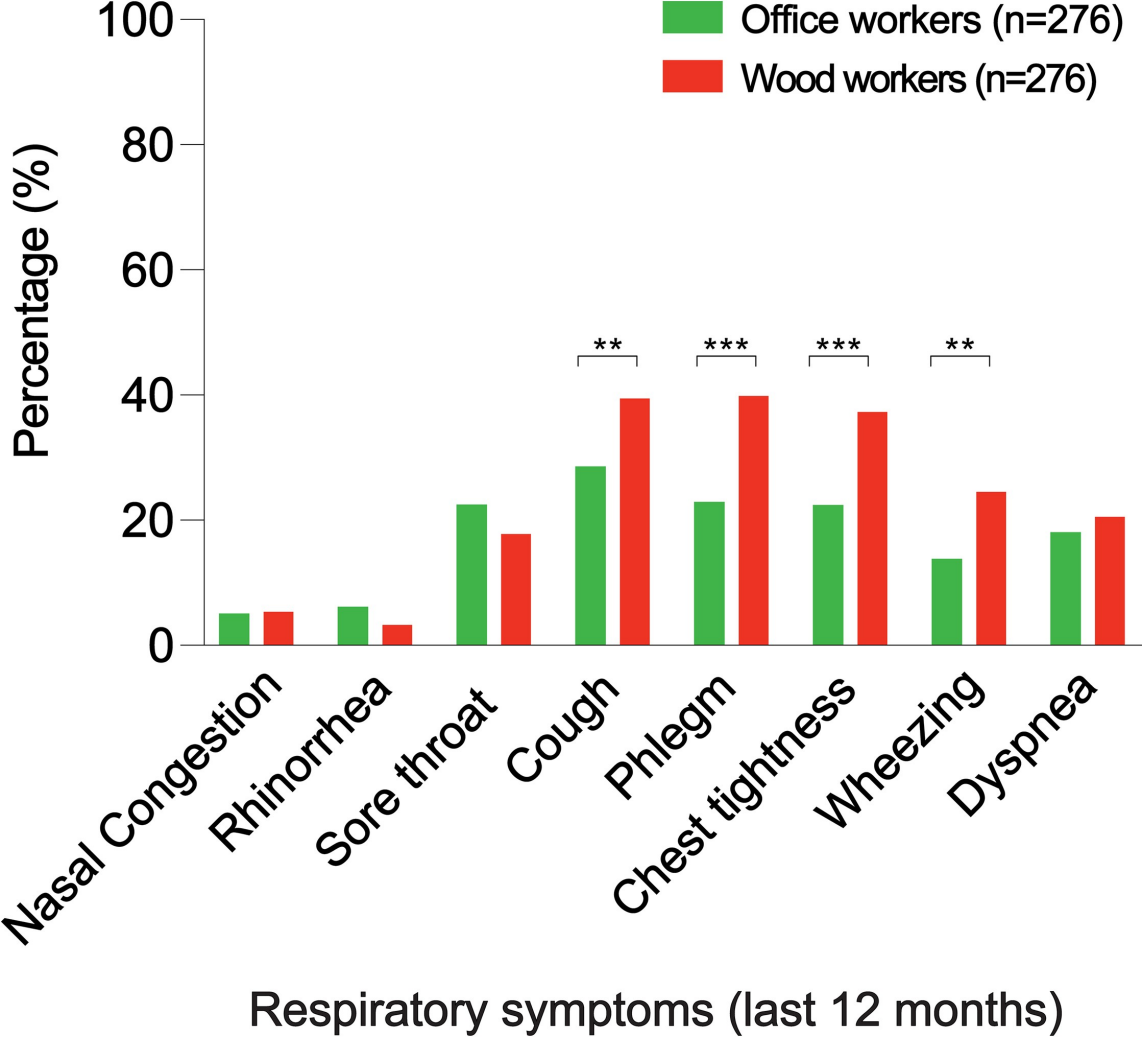
Twelve months period prevalence of respiratory symptoms, among wood workers and office workers. *p<0.05, **p<0.01, ***p<0.001.

Twelve-month period prevalence of nasal congestion, rhinorrhea, and sore throat, were not significantly different in wood workers that office workers (6.1% versus 5.8% for nasal congestion, p = 0.8575; 4.0% versus 6.9% for rhinorrhea, p = 0.1331; 18.5% versus 23.2% for sore throat, p = 0.1730) (Fig 2, and S1 Table).

### Lower respiratory tract symptoms

Twelve-month period prevalence of cough, phlegm, chest tightness, and wheezing, were significantly higher in wood workers than office workers (40.2% versus 29.3% for cough, p = 0.0073; 40.6% versus 23.6% for phlegm, p<0.0001; 38.0% versus 23.1% for chest tightness, p = 0.0001; 25.3% versus 14.5% for wheezing, p = 0.0014). Prevalence of dyspnea was not significantly different in wood workers than office workers (21.3% versus 18.8% for dyspnea, p = 0.4573) (Fig 2, and S1 Table).

Respiratory symptoms in 8.4% of wood workers, described as a “major medical illness”, however only 4% of office workers described respiratory symptoms as a “major medical illness” during the last twelve months (p = 0.0336). Similarly, 9.6% of wood workers reported, respiratory illness as a reason for quit/change job during the last twelve months, compared to 3.9% of office workers who had to quit/change their job due to respiratory involvements (p = 0.0036).

Moreover, a significant difference was found in the prevalence of respiratory symptoms including cough, phlegm, chest tightness, among the wood workers with duration of exposure 15 years and longer compared to those with shorter duration of exposure at the actual workplace (45.93% versus 30.77% for cough, p = 0.01280; 55.23% versus 16.35% for phlegm, p<0.0001; 43.60% versus 28.85% for chest tightness, p = 0.0144) (Table 3). In contrast such difference was not identified for respiratory symptom in the last 12 months among the office workers.

**Table 3:** Twelve months period prevalence of respiratory symptoms, among wood workers with duration of exposure at the actual workplace more and less than 15 years.

Respiratory symptoms in the last 12 months	Duration of employment < 15 years (n = 172)	Duration of employment ≥ 15 years (n = 104)	P-value*
Cough (n = 111)	79 (45.93%)	32 (30.77%)	0.01280
Phlegm (n = 112)	95 (55.23%)	17 (16.35%)	<0.0001
Chest tightness (n = 105)	75 (43.60%)	30 (28.85%)	0.0144
Wheezing (n = 70)	44 (25.58%)	26 (25.00%)	0.9143
Dyspnea (n = 59)	31 (18.02%)	28 (26.93%)	0.0805

* Chi-square test.

Spirometry test indicated with higher rate in wood workers than office workers (21.4% versus 5.4%, p<0.001). Spirometry test analysis in wood workers who agreed to have spirometry test (n = 48) revealed 25% abnormal readings.

The mean values of parameters measured in spirometry (FEV_1_ and FEV_1_/FVC) were significantly lower in the wood workers than office workers (85.20±8.34 versus 90.30±10.03 for %predicted FEV_1_, p = 0.023; 84.11±26.17 versus 88.24±18.02 for FEV_1_/FVC, p = 0.031) (Table 4).

**Table 4:** Spirometry test result.

Spirometry test	Wood workers (n = 48)	Office workers (n = 48)	P-value*
% predicted FEV_1_	85.20 ± 8.34	90.30 ±10.03	0.023
% predicted FVC	83.15 ± 21.27	87.43 ± 14.15	0.720
FEV_1_/FVC	84.11 ± 26.17	88.24 ± 18.02	0.031

Mean baseline values of spirometric parameters of study subjects.

*Compared by Independent-samples T-test.

The mean values of parameters measured in spirometry (FEV_1_ and FEV_1_/FVC) were significantly lower in the wood workers with duration of exposure 15 years and longer at the actual workplace than those with duration of exposure of shorter than 15 years (86.31±7.23 versus 83.02±6.16 for %predicted FEV_1_, p = 0.018; 85.04±25.24 versus 82.41±24.44 for FEV_1_/FVC, p = 0.041) (Table 5). However, such difference was not identified in the office workers. Obstructive lung disease was the most common pattern among wood workers with abnormal spirometry reading (71.4% obstructive pattern versus 28.6% restrictive pattern).

**Table 5:** Spirometry result.

Spirometry test	Wood workers (n = 48)		P-value*
	Duration of employment < 15 years (n = 32)	Duration of employment ≥ 15 years (n = 16)	
% predicted FEV_1_	86.31 ± 7.23	83.02 ± 6.16	0.018
% predicted FVC	83.39 ± 21.01	82.75 ± 20.87	0.832
FEV_1_/FVC	85.04 ± 25.24	82.41 ± 24.44	0.041

Mean baseline values of spirometric parameters among wood workers with duration of exposure at the actual workplace longer and shorter than 15 years

*Compared by Independent-samples T-test.

## Discussion

Occupational lung diseases are caused by pathologic responses of the airway to the working environment, leading to a substantial social and economic burden worldwide.

In addition to particle material, duration of exposure is an important factor determining host immune system response[12]. Experience has indicated that inflammatory response in the respiratory system can result in persistent symptomatic respiratory illnesses. While regular pulmonology visits with detailed physical examination is recommended in patients who chronically exposed to dust and particle in the working environment, a pulmonary function test should be considered in those with persistent respiratory symptom. Pulmonologists should be aware of the pattern and the ability to identify minimal changes in pulmonary function test, compared to the patient’s baseline, and incorporate this review routinely when searching for causes of respiratory illness in patient with occupational exposure.

Wood dust generated during sawing, carving, and drilling are made up of tiny particles of sub-5 μm which by-pass the filtering mechanism of the upper respiratory tract and penetrate into the lower respiratory system. Inflammation and subsequent scar tissue production in lungs results in comprised lung function and increased the risk of pulmonary diseases[5].

Previous studies investigating the association between occupational exposure to wood dust and the prevalence of chronic obstructive lung disease (COPD) displayed conflicting results[13–15]. Lung function change, described in wood workers has in several longitudinal studies been correlated to the level of organic dust exposure. In 1996, Noertjojo et al. examined 243 sawmill workers, who exposed to red cedar in 11 years follow up study, and their evaluations revealed that chronic exposure to western red cedar dust is associated with decline in lung function that is not due to presence of asthma[16].

Importance of industrial hygiene aimed to reduce the dust exposure among wood workers using technical improvements, including sharp cutting edge and local and general industrial ventilation systems like exhaust ventilation device and high efficiency particulate filters[17]. Protective clothing, goggles, and gloves are needed to reduce skin exposure to sawdust. Dust mask is also helpful in providing some forms of protection against inhalation of wood dust[18]. In addition, sawmill industries should be encouraged to purchase a gravimetric air sampling device in order to assess the concentration of sawdust within their workplace[19].

The present study aimed to determine the comparative study of the respiratory symptoms’ prevalence among wood workers. In summary, these data indicate that wood workers had significantly higher prevalence of respiratory symptoms, including the number of attacks of wheezing, cough, phlegm and chest tightness and changes in the spirometry test. To the best our knowledge this is first study conducted among large patient population, evaluated both upper and lower respiratory tract symptoms and consistently warranted the importance of regular follow up among patient who exposed to wood dust in the working environment.

A limitation of this study was the lack of enough data to support spirometric changes among wood workers. Though the results of this study suggest significantly lower FEV_1_ and FEV_1_/FVC ratios among wood workers, they are both within the normal range based on guidelines. Additional prospective studies relating changes in lung volumes among wood workers are needed to further establish the benefit of regular screening test to detect minimal deterioration in lung function in patients whom exposed to wood dust in the working environment.

In 2015 Staffieri et al. reported higher prevalence of nasal symptoms among woodworkers compared to control group (62% vs 41% respectively, p < 0.00001)[20], however in our study we were unable to detect such difference between wood workers and office workers. It could be explained by differences in the patient population were studied (e.g. different history of smoking) and/or because of the lack of objective tool in our study to precisely analyze any possible changes in upper respiratory airway. Further investigation is required by current available tools such as nasal cytology to detect influence of wood dust exposure on the upper respiratory airway[21].

Another limitation of the present study was the lack of ability to assess correlation between the severity of respiratory symptoms and the duration of exposure to wood dust among wood workers. We were unable to acquire more detailed history regarding the respiratory symptoms among our patient population given the cross-section nature of the current study. This requires prospective study with frequent clinical visit and monitoring to reduce the recall bias among participant.

Finally, education of employers and employees concerning the effects of wood dust on health and safety measures are essential for the success of occupational health programs.

## Conclusion

In conclusion findings suggested respiratory symptoms associated with work, are more prevalent among wood workers than office workers. Both the prevalence of respiratory symptoms and the reduction of spirometric parameters among wood workers correlated with the duration of the workplace exposure to wood dust. Additionally, obstructive changes on pulmonary function test is a dominant pathologic pattern in spirometry test among wood workers.

Our data revealed that workplace exposure to hardwood dust may compromise respiratory function, indicating the importance and the need for optimizing preventive measures in workplace to protect the respiratory health among exposed workers.

## Supporting information

S1 Form Respiratory symptoms questionnaire in Farsi. (PDF)

S2 Form Respiratory symptoms questionnaire in English. (DOCX)

S1 Table Twelve months period prevalence of respiratory symptoms, among wood workers and office workers. (PDF)

## References

[1] Hämäläinen P, Takala J, Kiat TB. Global estimates of occupational accidents and work-related illnesses 2017. World. 2017;2017:3–4.

[2] Sirajuddin A, Kanne JP. Occupational lung disease. Journal of thoracic imaging. 2009;24(4):310–20. 10.1097/RTI.0b013e3181c1a9b3 19935227

[3] Laney AS, Weissman DN. Respiratory diseases caused by coal mine dust. J Occup Environ Med. 2014;56 Suppl 10:S18–22. Epub 2014/10/07. 10.1097/JOM.0000000000000260 25285970 PMC4556416

[4] Adewole OO, Desalu OO, Nwogu KC, Adewole TO, Erhabor GE. Respiratory symptoms and lung function patterns in workers exposed to wood smoke and cooking oil fumes (mai suya) in Nigeria. Ann Med Health Sci Res. 2013;3(1):38–42. Epub 2013/05/02. 10.4103/2141-9248.109475 23634327 PMC3634221

[5] Mohan M, Aprajita, Panwar NK. Effect of wood dust on respiratory health status of carpenters. J Clin Diagn Res. 2013;7(8):1589–91. Epub 2013/10/03. 10.7860/JCDR/2013/5568.3231 24086847 PMC3782904

[6] Higuchi T. Biosynthesis and biodegradation of wood components. Orlando: Academic Press; 1985 xvi, 679 p. p.

[7] Bislimovska D, Petrovska S, Minov J. Respiratory Symptoms and Lung Function in Never-Smoking Male Workers Exposed To Hardwood Dust. Open Access Maced J Med Sci. 2015;3(3):500–5. Epub 2016/06/09. 10.3889/oamjms.2015.086 27275278 PMC4877847

[8] Schlunssen V, Jacobsen G, Erlandsen M, Mikkelsen AB, Schaumburg I, Sigsgaard T. Determinants of wood dust exposure in the Danish furniture industry—results from two cross-sectional studies 6 years apart. Ann Occup Hyg. 2008;52(4):227–38. Epub 2008/04/15. 10.1093/annhyg/men012 18407937 PMC2413102

[9] Ferris B. Epidemiology standardization project. II. Recommended respiratory disease questionnaires for use with adults and children in epidemiological research. Am Rev Respir Dis. 1978;118(6):7–53. 742764

[10] Variations in the prevalence of respiratory symptoms, self-reported asthma attacks, and use of asthma medication in the European Community Respiratory Health Survey (ECRHS). Eur Respir J. 1996;9(4):687–95. Epub 1996/04/01. 10.1183/09031936.96.09040687 . 8726932

[11] Standardization of Spirometry, 1994 Update. American Thoracic Society. Am J Respir Crit Care Med. 1995;152(3):1107–36. Epub 1995/09/01. 10.1164/ajrccm.152.3.7663792 . 7663792

[12] Ghosh T, Gangopadhyay S, Das B. Prevalence of respiratory symptoms and disorders among rice mill workers in India. Environ Health Prev Med. 2014;19(3):226–33. Epub 2014/03/13. 10.1007/s12199-014-0384-8 24609959 PMC4019758

[13] Shamssain MH. Pulmonary function and symptoms in workers exposed to wood dust. Thorax. 1992;47(2):84–7. Epub 1992/02/01. 10.1136/thx.47.2.84 1549828 PMC463576

[14] Borm PJ, Jetten M, Hidayat S, van de Burgh N, Leunissen P, Kant I, et al Respiratory symptoms, lung function, and nasal cellularity in Indonesian wood workers: a dose-response analysis. Occup Environ Med. 2002;59(5):338–44. Epub 2002/05/02. 10.1136/oem.59.5.338 11983850 PMC1740285

[15] Bolund ACS, Miller MR, Jacobsen GH, Sigsgaard T, Schlunssen V. New-onset COPD and Decline in Lung Function Among Wood Dust-Exposed Workers: Re-analysis of a 6-year Follow-up Study. Ann Work Expo Health. 2018;62(9):1064–76. Epub 2018/08/31. 10.1093/annweh/wxy075 . 30165410

[16] Noertjojo HK, Dimich-Ward H, Peelen S, Dittrick M, Kennedy SM, Chan-Yeung M. Western red cedar dust exposure and lung function: a dose-response relationship. Am J Respir Crit Care Med. 1996;154(4 Pt 1):968–73. Epub 1996/10/01. 10.1164/ajrccm.154.4.8887593 . 8887593

[17] Welling I, Lehtimaki M, Rautio S, Lahde T, Enbom S, Hynynen P, et al Wood dust particle and mass concentrations and filtration efficiency in sanding of wood materials. J Occup Environ Hyg. 2009;6(2):90–8. Epub 2008/12/10. 10.1080/15459620802623073 . 19065389

[18] Kwame O-B, Kusi E, Lawer E. Occupational hazards and safety practices: a concern among small scale sawmilling industries in Tamale Metropolis, Ghana. International Journal of Scientific & Technology Research. 2014;3(10):234–6.

[19] Lee T, Harper M, Slaven JE, Lee K, Rando RJ, Maples EH. Wood dust sampling: field evaluation of personal samplers when large particles are present. Annals of Occupational Hygiene. 2010;55(2):180–91. 10.1093/annhyg/meq075 21036895 PMC3037778

[20] Staffieri C, Lovato A, Aielli F, Bortoletto M, Giacomelli L, Carrieri M, et al Investigating nasal cytology as a potential tool for diagnosing occupational rhinitis in woodworkers. Int Forum Allergy Rhinol. 2015;5(9):814–9. Epub 2015/06/06. 10.1002/alr.21562 . 26046544

[21] Lovato A, Staffieri C, Ottaviano G, Cappellesso R, Giacomelli L, Bartolucci GB, et al Woodworkers and the inflammatory effects of softwood/hardwood dust: evidence from nasal cytology. Eur Arch Otorhinolaryngol. 2016;273(10):3195–200. Epub 2016/03/24. 10.1007/s00405-016-3989-2 . 27001257

