# Supplementary material for: Prevalence of respiratory symptoms and spirometric changes among non-smoker male wood workers
Source: PLoS One. 2020 Mar 18;15(3):e0224860. doi: 10.1371/journal.pone.0224860 (PMC7080227; doi:10.1371/journal.pone.0224860)
Supplement: S1 Form — (PDF) [file pone.0224860.s001.pdf]

با تشکر از مشارکت شما. همکاری شما جهت موفقیت این پروژه بسیار مهم میباشد

لطفا این پرسشنامه را تکمیل کنید. لطفا به سوالات این پرسشنامه با دقت و صادقانه پاسخ دهید

تمامی اطلاعات و پاسخ های شما محفوظ میباشد و بجز در تحقیق بکار نخواهد رفت

در صورت تمایل شما، پزشکتان از نتایج ازمایش شما مطلع خواهد شد
لطفا پاسخ های خود را با کشیدن دایره به دور پاسخ مد نظرتان مشخص کنید. در زمان ویزیت به شما جهت پر کردن فرم کمک خواهد شد

به عنوان مثال

آیا شما در ایران زندگی میکنید؟	بلی	خیر

تاریخ روز /ماه/سال

شماره بیمار

شغل

شماره مرکز پزشکی

جنس

تاریخ تولد/محل تولد

وزن

ادرس

تلفن

مصاحبه کننده/پزشک

۱. سابقه مصرف دخانیات(سیگار، سیگار برگ، قلیان، پایپ، غیره)

آیا تا به حال سیگار کشیده اید؟	بلی	خیر

(نه به معنی این است که شما کمتر از ۲۰ پاکت در زندگیتان سیگار کشیده اید)

آیا هم اکنون سیگاری هستید؟	بلی	خیر

۲. ایا دارای سابقه /تا کنون تشخیص بیماری مزمن ریه از جمله (و نه محدود به) هستید؟

اسم	بلی	خیر
بیماری انسدادی مزمن ریه	بلی	خیر
عفونت مزمن ریوی از جمله سل ریوی	بلی	خیر
فیبروز ریه	بلی	خیر
کانسر ریه/پلور	بلی	خیر
پنوموکونیوزیس	بلی	خیر

سارکوییدوزیس با درگیری ریه	بلی	خیر
ترومبو آمبولی ریه	بلی	خیر
افزایش فشار شریان ریوی	بلی	خیر
سایر موارد		

اگر پاسخ به سوالات یک و دو نه میباشد به سوال سه مراجعه کنی

سابقه پزشکی، سابقه جراحی، سابقه مصرف دارو

سابقه پزشکی			
دیابت نوع دو	بلی	خیر	
فشار خون	بلی	خیر	
چربی خون بالا	بلی	خیر	
بیماری ایسکمی قلبی	بلی	خیر	
درگیری عضلات /مفاصل	بلی	خیر	

سابقه جراحی			
ایا سابقه جراحی قبلی داشته اید	بلی	خیر	

سابقه دارو		

ایا طی سال گذشته دارو مصرف میکردید	بلی	خیر	

علاعم مرتبط با درگیری ریه

چهار. خس خس

ایا طی ۱۲ ماه گذشته شما خس خس سینه داشته اید؟	بلی	خیر

اگر پاسخ به سوال ‘نه’ میباشد به سراغ سوال ۵ بروید

ایا طی ۱۲ ماه گذشته در اغلب روز ها یا شب ها خس خس سینه داشته اید؟	بلی	خیر

ایا طی ۱۲ ماه گذشته در روز هایی که کار میکرده اید بیشتر از حد معمول خس خس سینه داشته اید ؟	بلی	خیر

ایا این خس خس سینه در زمانی که شما سرماخورده ویا عفونت ریوی نداشته بودید رخ داده بود؟	بلی	خیر

پنج سرفه

ایا طی ۱۲ ماه گذشته شما سرفه داشته اید؟<br>تمیزکردن گلو را به حساب نیاورید	بلی	خیر

اگر پاسخ به سوال ‘نه’ میباشد به سراغ سوال ۶ بروید

ایا طی ۱۲ ماه گذشته در اغلب روز ها یا شب ها سرفه داشته اید؟	بلی	خیر

ایا طی ۱۲ ماه گذشته در روز هایی که کار میکرده اید بیشتر از حد معمول سرفه داشته اید ؟	بلی	خیر

ایا این سرفه در زمانی که شما سرماخورده ویا عفونت ریوی نداشته بودید رخ داده بود؟	بلی	خیر

شش خلط

ایا طی ۱۲ ماه گذشته شما خلط داشته اید؟<br>موکوس و ترشحات پشت حلق را به حساب نیاورید	بلی	خیر

اگر پاسخ به سوال ‘نه’ میباشد به سراغ سوال ۷ بروید

ایا طی ۱۲ ماه گذشته در اغلب روز ها یا شب ها خلط داشته اید؟	بلی	خیر

ایا طی ۱۲ ماه گذشته در روز هایی که کار میکرده اید بیشتر از حد معمول خلط داشته اید ؟	بلی	خیر

ایا این خلط در زمانی که شما سرماخورده ویا عفونت ریوی نداشته بودید رخ داده بود؟	بلی	خیر

هفت تنگی سینه

ایا طی ۱۲ ماه گذشته شما تنگی سینه داشته اید؟	بلی	خیر

اگر پاسخ به سوال ‘نه’ میباشد به سراغ سوال۸ بروید

ایا طی ۱۲ ماه گذشته در اغلب روز ها یا شب ها تنگی سینه داشته اید؟	بلی	خیر

ایا طی ۱۲ ماه گذشته در روز هایی که کار میکرده اید بیشتر از حد معمول تنگی سینه داشته اید ؟	بلی	خیر

ایا این تنگی سینه در زمانی که شما سرماخورده ویا عفونت ریوی نداشته بودید رخ داده بود؟	بلی	خیر

هشت تنگی نفس

ایا طی ۱۲ ماه گذشته شما تنگی نفس داشته اید؟	بلی	خیر

اگر پاسخ به سوال 'نه' میباشد به سراغ سوال۹ بروید

ایا طی ۱۲ ماه گذشته هنگام با لا رفتن ازپله با سرعت معمول تنگی تنگی نفس داشته اید؟	بلی	خیر

ایا این تنگی نفس هنگامی که با هم سن و سالان خودتان با سرعت معمول فعالیت کنید روی میدهد؟	بلی	خیر

ایا طی ۱۲ ماه گذشته در روز هایی که کار میکرده اید بیشتر از حد معمول تنگی تنگی نفس داشته اید ؟	بلی	خیر

ایا طی ۱۲ ماه گذشته در روز هایی که کار میکرده اید بیشتر از حد معمول تنگی تنگی نفس داشته اید ؟	بلی	خیر

ایا این تنگی تنگی نفس در زمانی که شما سرماخورده ویا عفونت ریوی نداشته بودید رخ داده بود؟	بلی	خیر

این سوال توسط مصاحبه کننده پاسخ داده شود<br>ایا اختلال راه رفتن به عللی به غیر از درگیری قلبی ریوی است؟	بلی	خیر

نه احتقان بینی

ایا طی ۱۲ ماه گذشته شما احتقان بینی داشته اید؟	بلی	خیر

اگر پاسخ به سوال ‘نه’ میباشد به سراغ سوال۱۰ بروید

ایا طی ۱۲ ماه گذشته در روز هایی که کار میکرده اید بیشتر از حد معمول احتقان بینی داشته اید ؟	بلی	خیر

ایا این احتقان بینی در زمانی که شما سرماخورده ویا عفونت ریوی نداشته بودید رخ داده بود؟	بلی	خیر

ده ابریزش بینی

ایا طی ۱۲ ماه گذشته شما ابریزش بینی داشته اید؟	بلی	خیر

اگر پاسخ به سوال 'نه' میباشد به سراغ سوال۱۱ بروید

ایا طی ۱۲ ماه گذشته در روز هایی که کار میکرده اید بیشتر از حد معمول ابریزش بینی داشته اید ؟	بلی	خیر

ایا این ابریزش بینی در زمانی که شما سرماخورده ویا عفونت ریوی نداشته بودید رخ داده بود؟	بلی	خیر

یازده گلو درد

ایا طی ۱۲ ماه گذشته شما گلو درد داشته اید؟	بلی	خیر

ایا طی ۱۲ ماه گذشته در روز هایی که کار میکرده اید بیشتر از حد معمول گلو درد داشته اید ؟	بلی	خیر

ایا این گلو درد در زمانی که شما سرماخورده ویا عفونت ریوی نداشته بودید رخ داده بود؟	بلی	خیر

ایا شما درگیری ریه خود را'درگیری جدی سلامتی'خود میدانید؟	بلی	خیر

ایا در سال گذشته به عللی غیر از سرماخوردگی مجبور به مرخصی از محل کار به علت درگیری ریه شده بودید؟	بلی	خیر
