# Supplementary material for: Prevalence of respiratory symptoms and spirometric changes among non-smoker male wood workers
Source: PLoS One. 2020 Mar 18;15(3):e0224860. doi: 10.1371/journal.pone.0224860 (PMC7080227; doi:10.1371/journal.pone.0224860)
Supplement: S2 Form — (DOCX) [file pone.0224860.s002.docx]

Thank you for your willingness to participate. Your cooperation is very important to the success of the study.

This is a questionnaire you are asked to fill out. Please answer the questions as *frankly* and *accurately* as possible.

ALL INFORMATION OBTAINED IN THE SUTDY WILL BE KEPT CONFIDENTIAL AND USED FOR MEDICAL RESEARCH ONLY.

Your personal physician will be informed about the test results *if you desire*.

The questions can be answered by ***circling*** the best answer or by filling in a blank with a number or word. You will be helped with these questions at the time of your appointment.

Example:

| Do you live in Iran? | NO | YES |
| --- | --- | --- |

| Date (Day/ Month/ Year)  Patient ID #  Occupation:  Center ID #  Gender:  Date of birth/ Place of birth:  Weigh:  Height:  Body mass index (BMI: kg/m^2^)  Address:  Phone:  Interviewer/ Physician (First name/ Last name): | ------------------------------------------  ------------------------------------------  ------------------------------------------  ------------------------------------------  ------------------------------------------  ------------------------------------------  ------------------------------------------  ------------------------------------------  ------------------------------------------  ------------------------------------------  ------------------------------------------  ------------------------------------------ |
| --- | --- |

1. **History of smoking (cigarettes, cigars, hookahs, pipe tobacco, etc):**

| 1a. Have you ever smoked cigarettes?  (“NO” means less than 20 packs of cigarettes or 12 oz. of tobacco in a lifetime) | NO | YES |
| --- | --- | --- |

| 1b. Do you now smoke cigarettes? | NO | YES |
| --- | --- | --- |

1. **Do you have history of &/or have diagnosed with any chronic respiratory disease, including (but not limited to):**

| **Lung disorders** |  |  | **Note** |
| --- | --- | --- | --- |
| 2a. Asthma | NO | YES |  |
| 2b. Chronic obstructive pulmonary disease (COPD) | NO | YES |  |
| 2c. Chronic pulmonary infection, including pulmonary tuberculosis | NO | YES |  |
| 2d. Idiopathic pulmonary fibrosis | NO | YES |  |
| 2e. Lung/Pleural cancer | NO | YES |  |
| 2f. Pneumoconiosis | NO | YES |  |
| 2g. Sarcoidosis with lung involvement | NO | YES |  |
| 2h. Pulmonary thromboembolism | NO | YES |  |
| 2i. Pulmonary hypertension | NO | YES |  |
| Please indicate any other respiratory disease: | ------------------------------------------------------------- | | |

**If the answer to quest 1 & 2 is “NO”: GO TO QUESTION 3**

1. **Past medical history (PMH), past surgical history (PSH), and drug history: indicate which of the following you have had, or have at present. Circle “YES” or “NO” to each item.**

| **PMH** | **Note** (if “Yes” please provide name of medication) | | |
| --- | --- | --- | --- |
| 3a. Diabetes mellitus type-II | NO | YES |  |
| 3b. Hypertension (HTN) | NO | YES |  |
| 3c. Dyslipidemia | NO | YES |  |
| 3d. Ischemic heart disease (IHD) | NO | YES |  |
| 3e. Musculoskeletal disorders | NO | YES |  |

| **PSH** | Note (if “YES” please provide the details) | | |
| --- | --- | --- | --- |
| Do you have history of surgical intervention | NO | YES |  |

| **Drug history** | Note (if “YES” please provide the name/dose) | | |
| --- | --- | --- | --- |
| Have you taken any medication or drug during the past year | NO | YES |  |

**Respiratory related symptom questioner**

**Wheezing**

| 4a. Have you had wheezing or whistling in your chest at any time in the past 12 months? | NO | YES |
| --- | --- | --- |

**If “NO” go to question 5, if “YES”:**

| 4b. Have you had this wheezing or whistling on most days or nights during the past 12 months? | NO | YES |
| --- | --- | --- |

| 4c. Have you had this wheezing or whistling more than usual during daily work in the past 12 months? | NO | YES |
| --- | --- | --- |

| 4d. Have you had this wheezing or whistling when you ***did not*** have a cold/lung infection? | NO | YES |
| --- | --- | --- |

**Cough**

| 5a. Have you had cough at any time in the past 12 months?  **Do not** count clearing of throat. | NO | YES |
| --- | --- | --- |

**If “NO” go to question 6, if “YES”:**

| 5b. Have you found that you *usually* cough during the past 12 months?  **Do not** count occasional cough. | NO | YES |
| --- | --- | --- |

| 5c. Have you had this cough on most days or nights for 3 months during the past 12 months? | NO | YES |
| --- | --- | --- |

| 5d. Have you had this cough more than usual during daily work in the past 12 months? | NO | YES |
| --- | --- | --- |

| 5e. Have you had this cough when you ***did not*** have a cold/lung infection? | NO | YES |
| --- | --- | --- |

**Phlegm**

| 6a. Have you brought up any phlegm from you chest at any time in the past 12 months?  **Do not** count mucus from the throat/back of your nose. | NO | YES |
| --- | --- | --- |

**If “NO” go to question 7, if “YES”:**

| 6b. Have you had this phlegm on most days or nights for 3 months during the past 12 months? | NO | YES |
| --- | --- | --- |

| 6c. Have you had this phlegm more than usual during daily work in the past 12 months? | NO | YES |
| --- | --- | --- |

| 6d. Have you had this phlegm when you ***did not*** have a cold/lung infection? | NO | YES |
| --- | --- | --- |

**Chest tightness**

| 7a. Have you had chest tightness at any time in the past 12 months? | NO | YES |
| --- | --- | --- |

**If “NO” go to question 8, if “YES”:**

| 7b. Have you had this chest tightness more than usual during daily work in the past 12 months? | NO | YES |
| --- | --- | --- |

| 7c. Have you had this chest tightness when you ***did not*** have a cold/lung infection? | NO | YES |
| --- | --- | --- |

**Dyspnea**

| 8a. Have you had breathlessness at any time in the past 12 months? | NO | YES |
| --- | --- | --- |

**If “NO” go to question 9, if “YES”:**

| 8b. Have you had this breathlessness with going up one flight of stairs at your normal pace? | NO | YES |
| --- | --- | --- |

| 8c. Have you had this breathlessness when you walk with other people of your own age on the  flat at a normal pace? | NO | YES |
| --- | --- | --- |

| 8d. Have you had this breathlessness more than usual during daily work in the past 12 months? | NO | YES |
| --- | --- | --- |

| 8e. Have you had this breathlessness when you ***did not*** have a cold/lung infection? | NO | YES |
| --- | --- | --- |

| This question needs to be answered by **interviewer/physician**: Does the subject have  difficulty walking because of a condition other than due to cardiopulmonary disease? | NO | YES |
| --- | --- | --- |

**Nasal congestion**

| 9a. Have you had nasal congestion at any time in the past 12 months? | NO | YES |
| --- | --- | --- |

**If “NO” go to question 10, if “YES”:**

| 9b. Have you had this nasal congestion more than usual during daily work in the past 12 months? | NO | YES |
| --- | --- | --- |

| 9c. Have you had this nasal congestion when you ***did not*** have a cold/lung infection? | NO | YES |
| --- | --- | --- |

**Rhinorrhea**

| 10a. Have you had runny nose (rhinorrhea) at any time in the past 12 months? | NO | YES |
| --- | --- | --- |

**If “NO” go to question 11, if “YES”:**

| 10b. Have you had this rhinorrhea more than usual during daily work in the past 12 months? | NO | YES |
| --- | --- | --- |

| 10c. Have you had this runny nose when you ***did not*** have a cold/lung infection? | NO | YES |
| --- | --- | --- |

**Sore throat**

| 11a. Have you had sore throat at any time in the past 12 months? | NO | YES |
| --- | --- | --- |

| 11b. Have you had this sore throat more than usual during daily work in the past 12 months? | NO | YES |
| --- | --- | --- |

| 11c. Have you had this sore throat when you ***did not*** have a cold/lung infection? | NO | YES |
| --- | --- | --- |

| 12. Do you consider your respiratory system involvement as your “Major medical illness”? | NO | YES |
| --- | --- | --- |

| 13. Have you taken a day/or more days off from your work, due to your respiratory system illness?  (when you ***did not*** have a cold/lung infection) | NO | YES |
| --- | --- | --- |
