# Supplementary material for: Prevalence of respiratory symptoms and spirometric changes among non-smoker male wood workers
Source: PLoS One. 2020 Mar 18;15(3):e0224860. doi: 10.1371/journal.pone.0224860 (PMC7080227; doi:10.1371/journal.pone.0224860)
Supplement: S1 Table — (PDF) [file pone.0224860.s003.pdf]

| Respiratory symptoms in the last 12 months | Wood workers (n=276) | Office workers (n=276) | P-value* |
|---|---|---|---|
| Nasal congestion | 17 (6.1%) | 16 (5.8%) | 0.8575 |
| Rhinorrhea | 11 (4.0%) | 19 (6.9%) | 0.1331 |
| Sore/scratchy throat | 51 (18.5%) | 64 (23.2%) | 0.1730 |
| Cough | 111 (40.2%) | 81 (29.3%%) | 0.0073 |
| Phlegm | 112 (40.6%) | 66 (23.6%) | <0.0001 |
| Chest tightness | 105 (38.0%) | 64 (23.1%) | 0.0001 |
| Wheezing | 70 (25.3%) | 40 (14.5%) | 0.0014 |
| Dyspnea | 59 (21.3%) | 52 (18.8%) | 0.4573 |

* Chi-square test.
